# Comparison of infectivity and spread between HSV-1 and HSV-2 based oncolytic viruses on tumor cells with different receptor expression profiles

**DOI:** 10.18632/oncotarget.25096

**Published:** 2018-04-20

**Authors:** Xinping Fu, Lihua Tao, Pin-Yi Wang, Timothy P. Cripe, Xiaoliu Zhang

**Affiliations:** ^1^ Department of Biology and Biochemistry, University of Houston, Houston, Texas, USA; ^2^ Center for Nuclear Receptors and Cell Signaling, University of Houston, Houston, Texas, USA; ^3^ Center for Childhood Cancer and Blood Diseases, Nationwide Children's Hospital, Columbus, Ohio, USA; ^4^ Department of Pediatrics, The Ohio State University, Columbus, Ohio, USA

**Keywords:** oncolytic herpes simplex virus, virotherapy, virus receptors, virus entry, infectivity

## Abstract

Herpes simplex virus (HSV) is one of the many viruses that have been modified or adapted for oncolytic purposes. There are two serotypes of HSV, HSV-1 and HSV-2. The majority of oncolytic HSVs, including T-VEC which has recently been approved by the US Food and Drug Administration (FDA) for clinical use in treating late stage melanoma patients, are derived from HSV-1. Recently, we and others have developed several HSV-2 based oncolytic viruses. During our *in vitro* characterization of oncolytic viruses developed from both serotypes (Baco-1 from HSV-1 and FusOn-H2 from HSV-2), we noticed there is a subpopulation of cancer cells in which both viruses could infect but only FusOn-H2 could spread from cell to cell on monolayers. This observation prompted us to investigate the virus receptor expression profiles in these and other tumor cells. Our data show the following: 1) This subpopulation of tumor cells only express nectin-2, not the other two major receptors (HVEM or nectin-1). 2) Baco-1 grows to a higher titer than FusOn-H2 in this subpopulation of tumor cells, but the latter kills these tumor cells more efficiently than the former. 3) FusOn-H2 is effective at treating tumors formed from these tumor cells while Baco-1 is completely ineffective. Our results suggest that this subpopulation of tumor cells may be intrinsically resistant to the therapeutic effect of a HSV-1 based oncolytic virus but they remain sensitive to a HSV-2 based virotherapy.

## INTRODUCTION

Herpes simplex virus (HSV) is one of the many human and animal viruses that have been modified or adapted for oncolytic purpose. Several intrinsic properties of HSV make it an attractive candidate as an oncolytic agent. First, lytic infection by HSV usually kills target cells much more rapidly than infection by other DNA viruses. For example, HSV can form visible plaques in cultured cells in only 2 days, in contrast to 7 to 9 days for adenovirus. *in vitro* studies have also shown that at a multiplicity of infection (MOI) of 0.01, HSV can kill almost 100% of cultured cancer cells in 2 days [1], while a much higher dose or a longer infection time is needed to achieve equivalent cell killing with a conditionally replicating adenovirus [2]. Rapid replication and spreading among target cells appear to be vital properties allowing a virus to execute its full oncolytic potential *in vivo*, as the body's immune mechanism may be more likely to restrict the spread of slower growing viruses. Second, HSV has a wide tropism and oncolytic viruses derived from it can be applied therapeutically to many different types of tumors. In principle, this property should protect against the rapid development of resistance to virotherapy using HSV in contrast to other oncolytic viruses such as those derived from adenoviruses. Third, effective anti-HSV medications such as acyclovir and famciclovir are readily available as safety measures in the event of undesired infection or toxicity from the virus. Finally, the risk of introducing an insertional mutation during HSV oncolytic therapy appears minimal because HSVs rarely integrate into cellular DNA.

There are two serotypes of HSV, HSV-1 and HSV-2. The majority of oncolytic HSVs, including T-VEC which has recently been approved by the US Food and Drug Administration (FDA) for clinical use in treating late stage melanoma patients, are derived from HSV-1 [3]. Recently, several HSV-2 based oncolytic viruses have also been developed, some of which will likely enter clinical trials in the near future [4–6]. Although HSV-1 and HSV-2 share approximately 50% homology in their genome sequences and many of the genes are co-liner, there are also substantial differences between these two viruses. One area of potential differences between these two viruses that is pertinent to oncolytic virotherapy but has not yet been fully elucidated, is their infectivity and the ability to spread from cell to cell within tumors of different tissue origins.

During HSV infection, viral particles initially attach to host cells through the binding of the viral envelope glycoproteins gB and/or gC to heparan sulfate proteoglycans (HSPG) on the cell surface [7]. However, this binding is neither essential nor sufficient for viral entry. Following this initial binding, another viral envelop glycoprotein, gD, engages one of several entry receptors [8]. The receptor binding by gD triggers fusion of the viral envelope with the cell membrane and then entry of the viral nucleocapsid and tegument into the cell cytoplasm. This envelope-membrane fusion requires the participation of three additional viral envelope glycoproteins, gB and gH-gL [8, 9]. So far, four gD binding receptors have been identified [8]. They include HVEM (herpesvirus entry mediator), a member of the TNF receptor family [10]; nectin-1 and nectin-2, members of the immunoglobulin superfamily [11, 12]; and specific sites in heparan sulfate generated by certain 3-*O*-sulfotransferases [13]. The gDs of HSV-1 and HSV-2 bind to these receptors with somewhat different preferences. HVEM and nectin-1 are efficient entry receptors for both HSV-1 and HSV-2. However, nectin-2 has been found to serve as entry receptor mainly for HSV-2, and conversely, 3-*O*-sulfate-modified heparan sulfate has been found to only facilitate the entry of HSV-1 [12, 13]. It is believed that these same receptors are used by the viruses for cell to cell spreading within tissues after initial infection.

The crux of an oncolytic virus is its ability to conditionally replicate and spread within tumor tissues. During our *in vitro* characterization of oncolytic viruses derived from HSV-1 (Baco-1) and HSV-2 (FusOn-H2), we encountered some tumor cells in which both viruses could infect but only FusOn-H2 was able to spread from cell to cell for plaque formation. This prompted us to fully examine the receptor expression profile in these and other tumor cells. Our data show that many of these tumor cells only express nectin-2 but not HVEM or nectin-1, indicating that these subpopulation of tumor cells are intrinsically resistant to the killing effect of the HSV-1 based oncolytic virus but remain permissive to the oncolytic effect of the HSV-2 based oncolytic virus. For other tumor cells that express either HVEM or nectin-1 alone or both of them, we found similar oncolytic effect of viruses derived from both HSV serotypes. Our data thus indicate that in future clinical applications, patients who are intrinsically resistant or have developed resistance to HSV-1 based oncolytic viruses may still be treatable by virotherapy from HSV-2 based oncolytic viruses.

## RESULTS

### Receptor expression profile of tumor cells in which FusOn-H2 but not Baco-1 could spread from cell to cell

**Figure 1:**
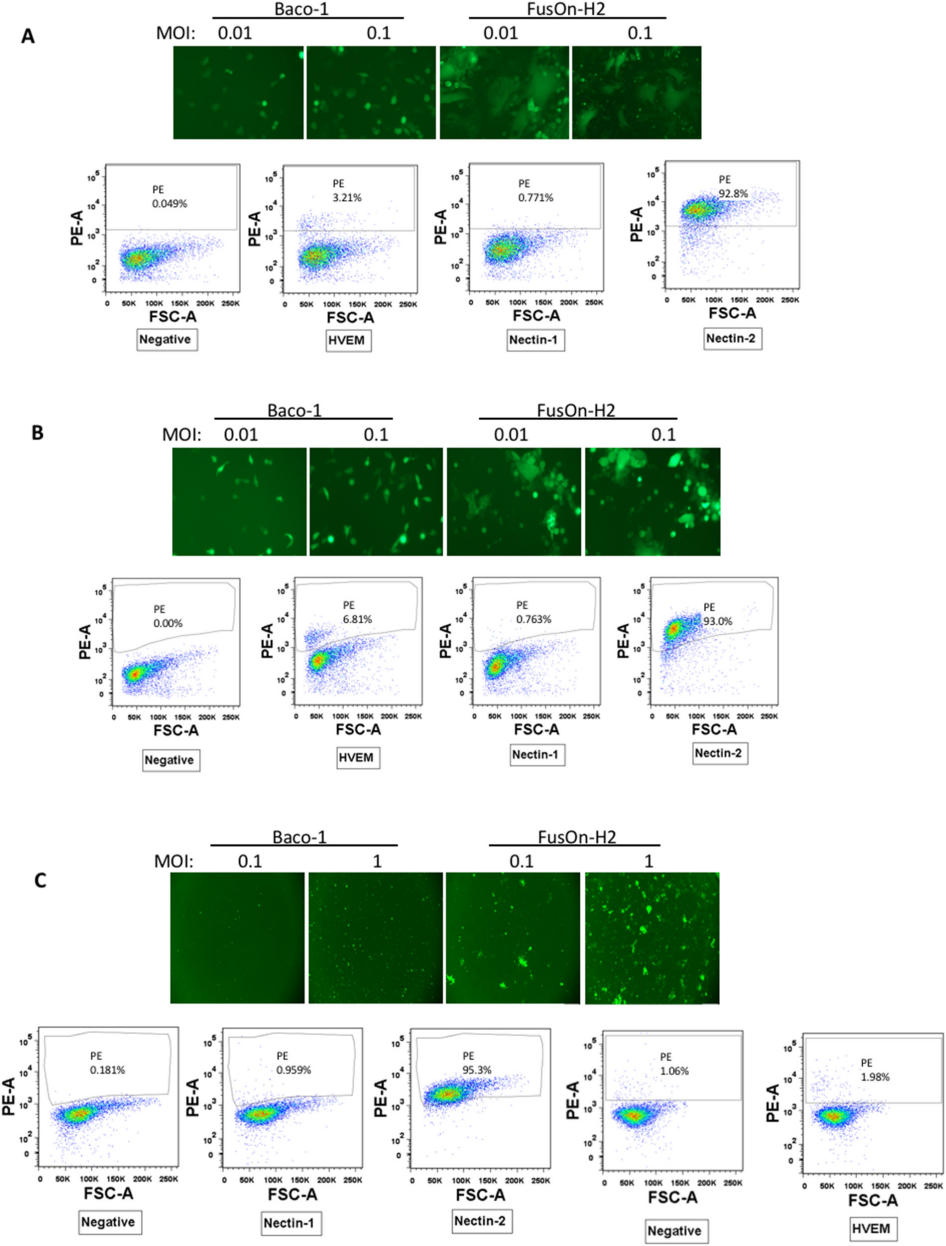
Characterization of a subpopulation of tumor cells that only support cell-cell spread of HSV-2 based but not HSV-1 based oncolytic virus **(A)** Hela cells (a human cervical cancer cell line). **(B)** EC9706 cells (a human esophageal carcinoma cell line). **(C)** A375 cells (a human melanoma cell line). The top panels show a typical field under fluorescent microscopy of cells infected by Baco-1 or FusOn-H2 at the indicated multiplicity of infection for 24 h. Original magnifications: 200X for A and B, 100X for C. The bottom panels show the results of flow cytometry for each of the three gD binding receptors. The flow cytometry for detection of nectin-1, 2 and HVEM in A375 cells were done in separate experiments.

**Figure 2:**
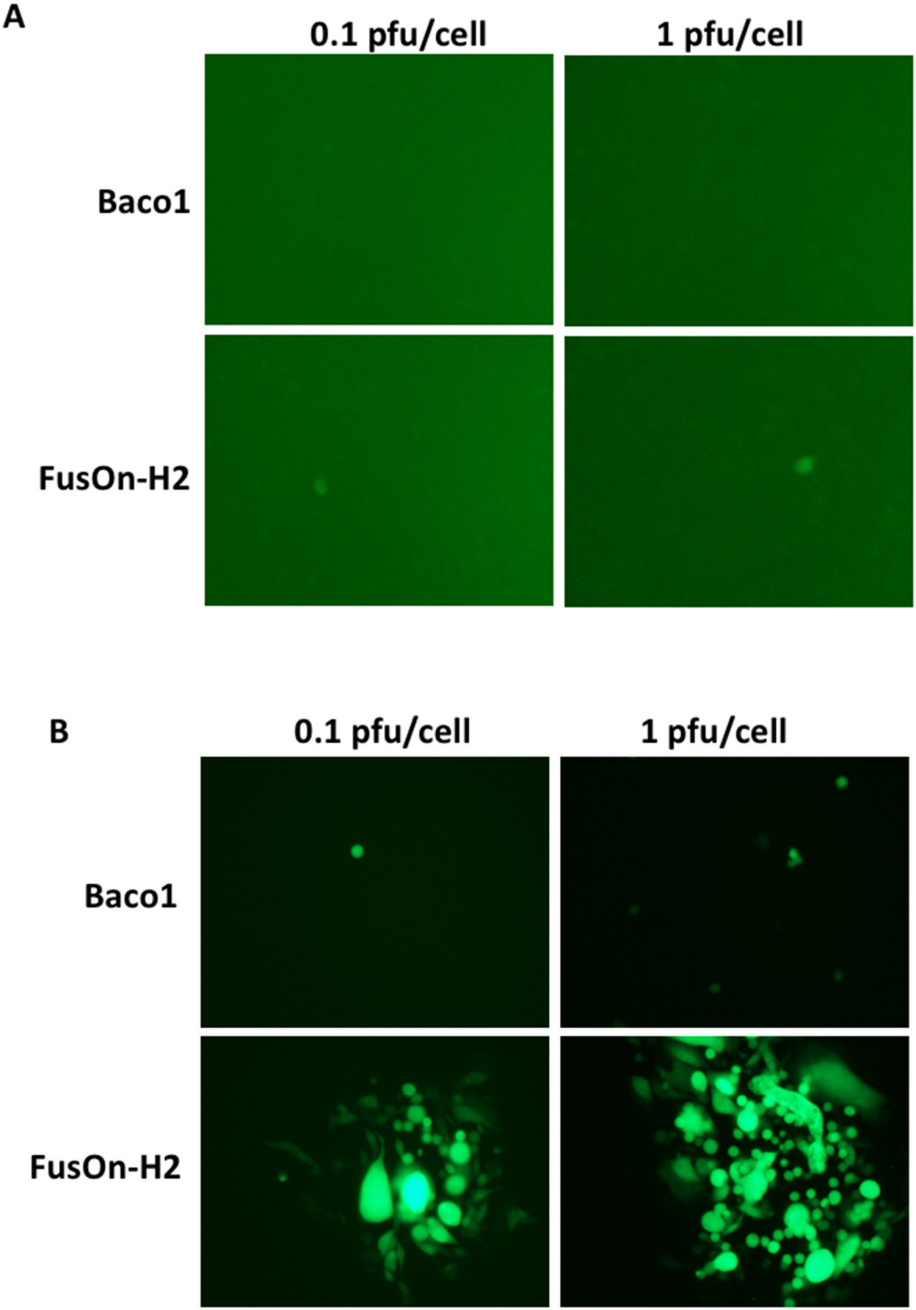
Transduction of nectin-2 gene into CHO-K1 cells allow the entry of both Baco-1 and FusOn-H2 but only enables the later to spread cell-cell CHO-K1 **(A)** and CHO-N2 **(B)** cells were infected with either Baco-1 or FusOn-H2 at the indicated MOI. Shown are a typical field from each well 48 h after virus infection. Original magnification: 200X.

**Figure 3:**
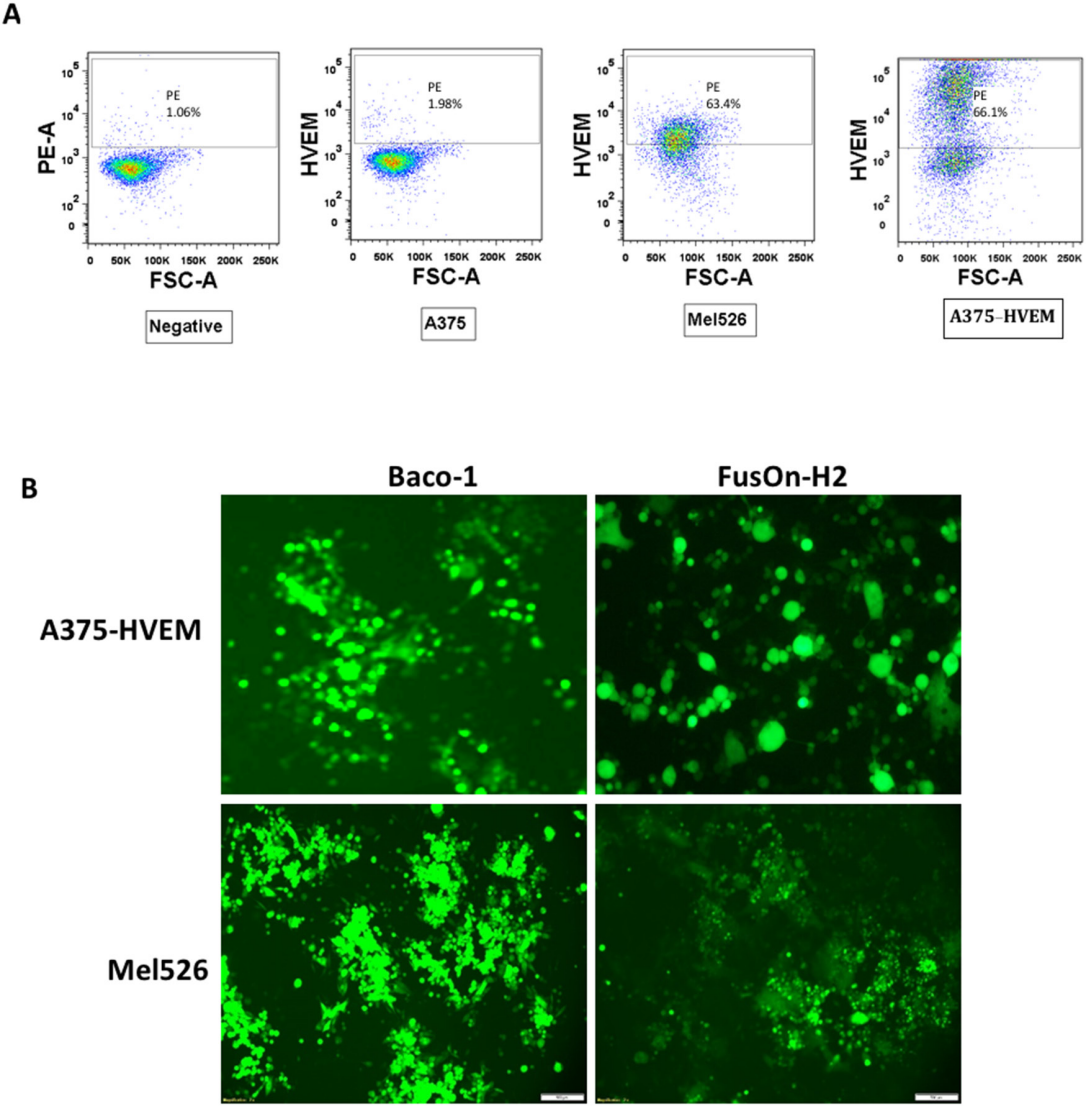
Transduction of HVEM to A375 cells allow both Baco-1 and FusOn-H2 entry and spread **(A)** Flow cytometry detection of HVEM expression in A375-HVEM and Mel526 cells. **(B)** Visualization of infection and spread of Baco-1 and FusOn-H2 in A375-HVEM and Mel526 cells after infecting the cells with the viruses at 0.1 pfu/cell. Shown are a typical field from each well at 24 h after infection. Original magnification: 200X (for A375-HVEM cells) and 100X for Mel526 cells.

In our efforts at developing HSV-based virotherapy, we constructed oncolytic viruses from both HSV-1 and HSV-2. Baco-1 was derived from HSV-1 and was similarly constructed as T-VEC. It has both copies of the *ICP34.5* gene deleted. It also contains a copy of the green fluorescent protein (*GFP*) gene for the purpose of tracking virus infection and spreading [14]. FusOn-H2 was derived from HSV-2 by deleting the N-terminal region of the *ICP10* gene [4]. Like Baco-1, it also carries the *GFP* gene. During our *in vitro* characterization, we encountered several tumor cell lines in which FusOn-H2, but not Baco-1, could initiate plaque formation. A viral plaque forms with an initially infected cell, from which the released progeny viruses spread to the surrounding cells. To determine if differential receptor expression contributed to this discrepancy, we analyzed these tumor cells by flow cytometry for cell surface expression of three known HSV receptors, HVEM, nectin-1 and nectin-2. We did not determine the expression status of the fourth receptor, which is a uniquely modified form (3-*O*-sulfate-modification) of heparin sulfate as its role in the infection of human target cells remained unclear. The flow cytometry results, together with the results from GFP visualization on virus infection and cell to cell spread, are shown in Figure 1. These experiments revealed several interesting findings. First, the flow cytometry analysis (the histograms on the bottom part on each of A. B and C) show that these cells either completely lack or express very little HVEM or nectin-1, the two major receptors used by both HSV-1 and HSV-2 for entry. The positive cells stained for nectin-1 in these cells are 0.77%, 0.76% and 0.95%, respectively. Instead, they all express a high level of nectin-2 and the positive cells stained for nectin-1 in these cells are 92.8%, 93.0% and 95.3%, respectively. Second, the GFP images on the top panels show that the HSV-1 derived Baco-1 seems to enter these cells at a similar efficiency as the HSV-2 derived FusOn-H2. This is despite the fact that nectin-2 has been found to be only used by HSV-2 for entry. Because HSV-1 does not typically utilize nectin-2 for entry (as confirmed in Figure 2 below), these cells may express other unknown receptors such as 3-*O*-S heparin sulfate. However, the GFP images on the top panels demonstrate that, unlike FusOn-H2, Baco-1 did not show any sign of significant spread following infection. In contrast, FusOn-H2 was able to spread to the surrounding tumor cells either through the syncytia formation (in both Hela and EC9706 cells) or through the conventional cell-cell transmission (in A375 cells). Nevertheless, the magnitude of spreading seems to be less extensive than we normally saw with some other tumor cells (e.g., as compared to the result in Figure 3B which shows a much greater extent of virus spreading in Mel526 and A375-HVEM cells with the same 24 h incubation period).

To test if nectin-2 alone allows the entry of both Baco-1 and FusOn-H2 into tumor cells and it additionally allows cell-cell spreading of FusOn-H2, we infected CHO-N2 cells with both viruses. CHO-N2 was derived from CHO-B, which was established by transducing the nectin-2 gene into CHO-K1 cells. As CHO-K1 does not express any of the four known HSV receptors and is resistant to HSV infection, nectin-2 would be the sole receptor for virus entry in CHO-N2 cells. The results in Figure 2A showed that the parental CHO-K1 cells were completely resistant to the infection of both Baco-1 and only sparsely infected by FusOn-H2, probably due to the presence of a yet unidentified endogenous receptor for HSV-2 in this cell [10]. In contrast, CHO-N2 allows only sparsely entry of both viruses (Figure 2B). Similar to the results shown in Figure 1, FusOn-H2 but not Baco-1 could spread from cell to cell after entry. These results thus confirm that nectin-2 alone can allow the cellular entry of both Baco-1 and FusOn-H2, but it only enables the HSV-2 based oncolytic virus to spread from cell to cell on the cell monolayer.

In the next experiment, we transduced HVEM gene into A375 to generate A375-HVEM, and then examined the infectivity and spread of both Baco-1 and FusOn-H2. We included another human melanoma cell line (Mel526) that expresses HVEM in this experiment as well. Flow cytometry analysis confirmed that both Mel526 and A375-HVEM cells express HVEM, with positive staining at 63.4% and 66.1%, respectively (Figure 3A). Both cells could be readily infected by Baco-1 and FusOn-H2 (Figure 3B), and most importantly, both Baco-1 and FusOn-H2 were found to be able to spread in these cells. These data thus suggest that there is nothing in these cells that intrinsically prevents Baco-1 from cell to cell spreading other than lack of an appropriate receptor, and that HVEM (or probably nectin-1) is needed for HSV-1 based oncolytic virus to exhibit cell-cell spread.

### Virus yield of Baco-1 and FusOn-H2 is independent of receptor expression profile

**Figure 4:**
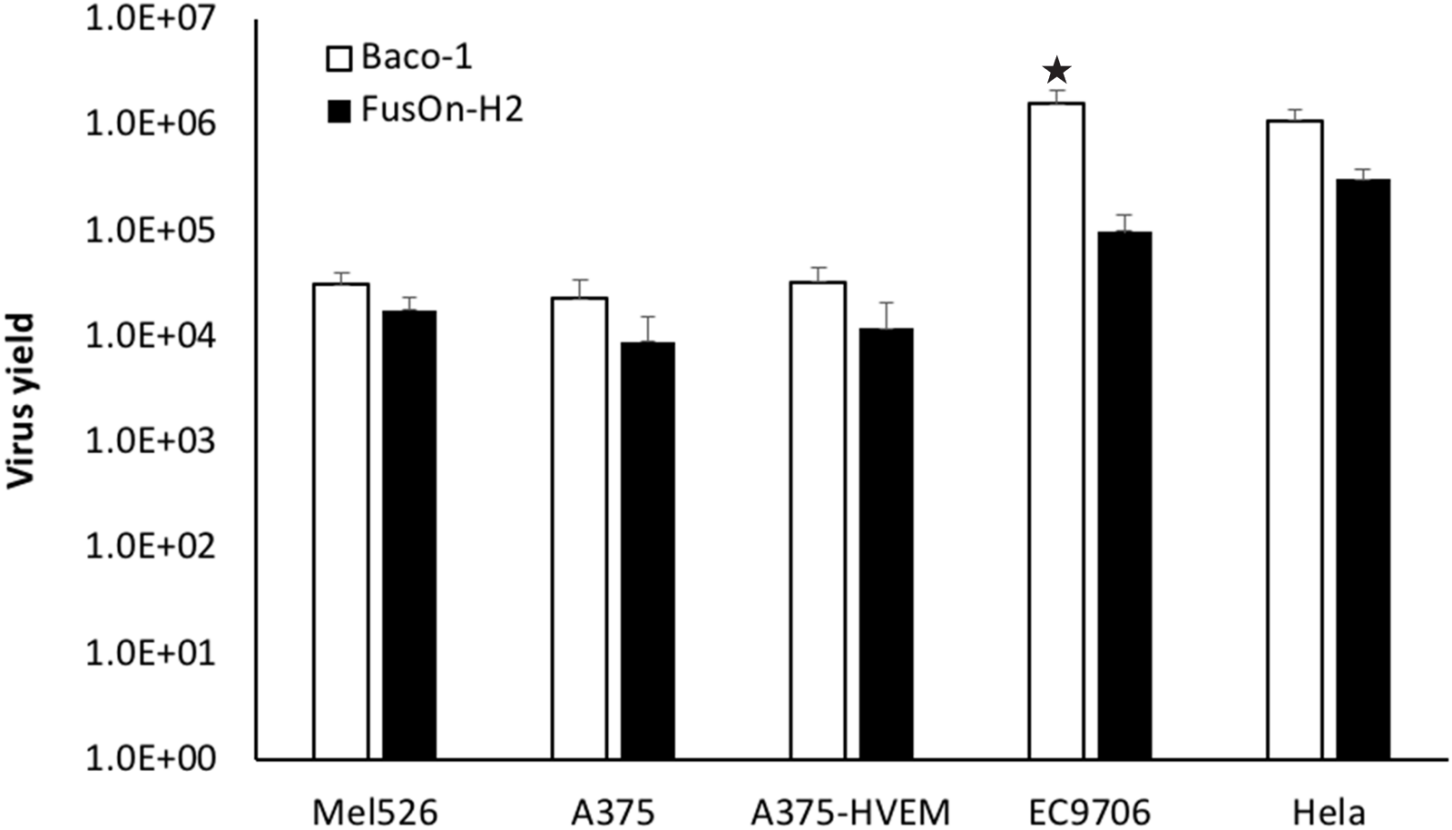
Virus yield of Baco-1 and FusOn-H2 in tumor cell lines that express different receptor profile Cells were infected with 1 pfu/cell of Baco-1 or FusOn-H2 for one hour before the infectants were removed and cells washed. The cells were cultured for 24 h before they were harvested and lysed for determination of virus yield by plaque assay. ★p<0.05 as compared with FusOn-H2.

Next, we compared the virus yield in this subpopulation of tumor cells after infection with Baco-1 and FusOn-H2 to determine how or if it is also impacted by the different receptor expression profile. We included some other tumor cells with different receptor expression profile in this experiment as well. Cells were infected with either Baco-1 or FusOn-H2 at a relatively high dose (1pfu/cell) since this subpopulation of tumor cells were less permissive to infection by both viruses. Cells were harvested 24 h later for determination of virus yield by plaque assay. The results in Figure 4 show that, in general, the virus yield from Baco-1 is higher than that of FusOn-H2. It seems to be true for this subpopulation of tumor cells that only express nectin-2 receptor, despite the inability of Baco-1 to show cell to cell spread in these cells (A375, Hela and EC9706). In particular, the yield of Baco-1 is more than a log higher than that of FusOn-H2 in EC9706 cells. These data suggest that nectin-2 expression alone only impacts the cell to cell spread of Baco-1 but has little or no impact on its replication after it has entered tumor cells.

### Tumor cell killing activity of Baco-1 and FusOn-H2 in tumor cells expressing different receptor profile

**Figure 5:**
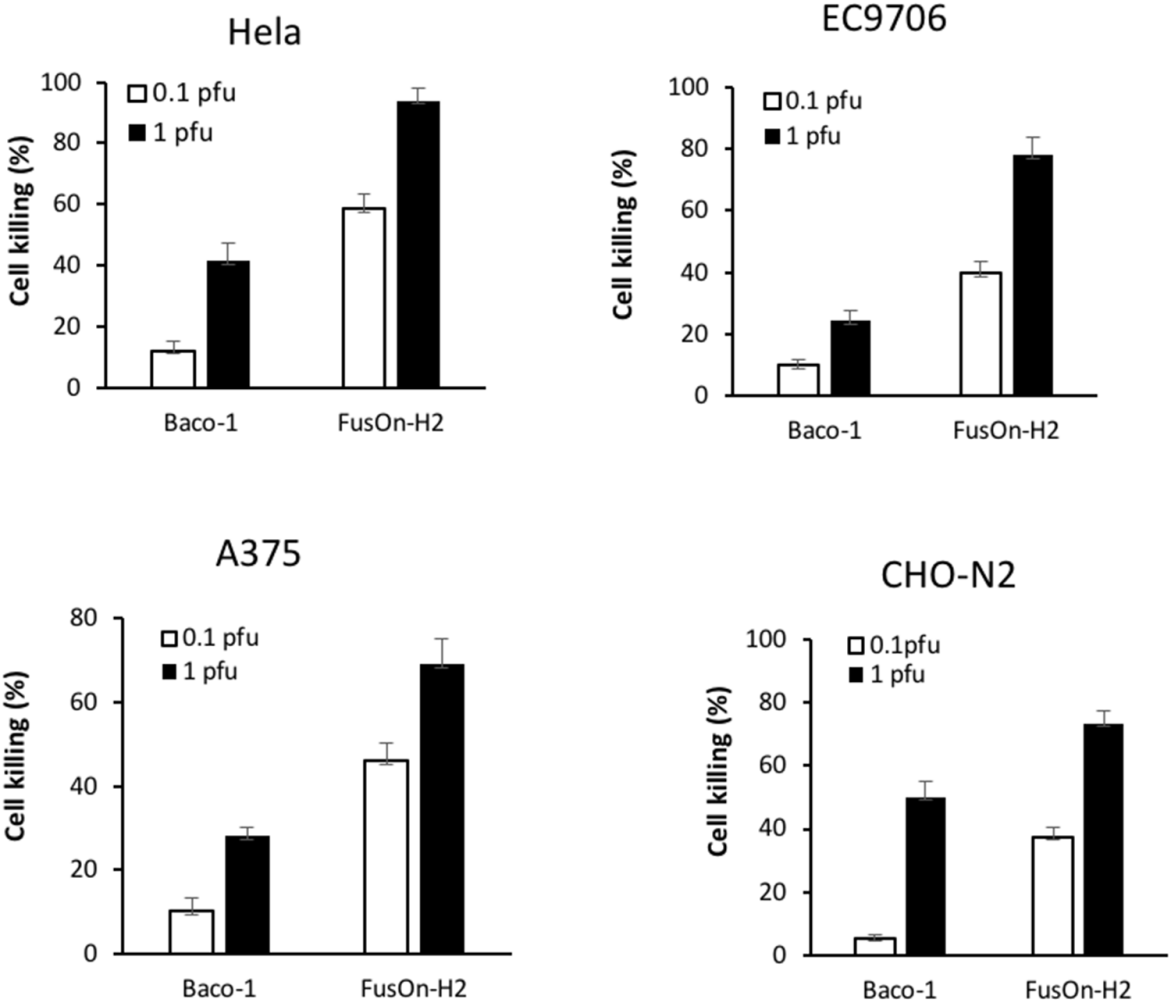
Comparison of killing activity between Baco-1 and FusOn-H2 on tumor cells that express nectin-2 but not HVEM or nection-1 Cells were infected with either Baco-1 or FusOn-H2 at either 0.1 or 1 pfu/cell, or mock infected. Cells were harvested 48 h later for quantification of viable cells. The percentage of cell killing was calculated by the following formula: (living cells of mock-infected well – living cells of infected well)/living cells of mock infected well x100. p<0.05 when comparing FusOn-H2 with Baco-1 at both MOIs in all the four cells.

The direct oncolytic effect of virotherapy primarily derives from the ability of the virus to initially infect tumor cells and then to spread to the surrounding tumor cells. The results in Figures 1 and 4 show a dichotomous outcome for Baco-1 and FusOn-H2 when evaluated in tumor cells that only express nectin-2 but not HVEM or nectin-1, i.e., Baco-1 failed to show cell-cell spreading but was able to produce high virus yield in these tumor cells while it is almost the opposite for FusOn-H2. To determine how this translates to tumor cell killing, we infected Hela, EC9706, A375 and CHO-N2 tumor cells with Baco-1 and FusOn-H2 at 0.1 or 1 pfu/cell. All these cells express low or no HVEM and nectin-1, but express high level of nectin-2. Cell viability was determined 48 h after virus infection. The results in Figure 5 show that FusOn-H2 infection led to significantly more tumor cell killing than Baco-1 in all these tumor cells at both multiplicities of infection, despite its lower virus yield in these cells. These results suggest that the ability of an oncolytic HSV to spread among tumor cells probably plays a more important role than the virus yield in contributing to the killing of tumor cells. Noticeably, FusOn-H2 was unable to induce a clear syncytial phenotype in one of these 4 cell lines, A375 (Figure 1). As such, at least in this tumor cell, the superior tumor cell killing by FusOn-H2 over Baco-1 was not due to its syncytial phenotype.

### *In vivo* evaluation of oncolytic effect of Baco-1 and FusOn-H2 against tumor established from one of the tumor cells that only express nectin-2 but not HVEM or nection-1

**Figure 6:**
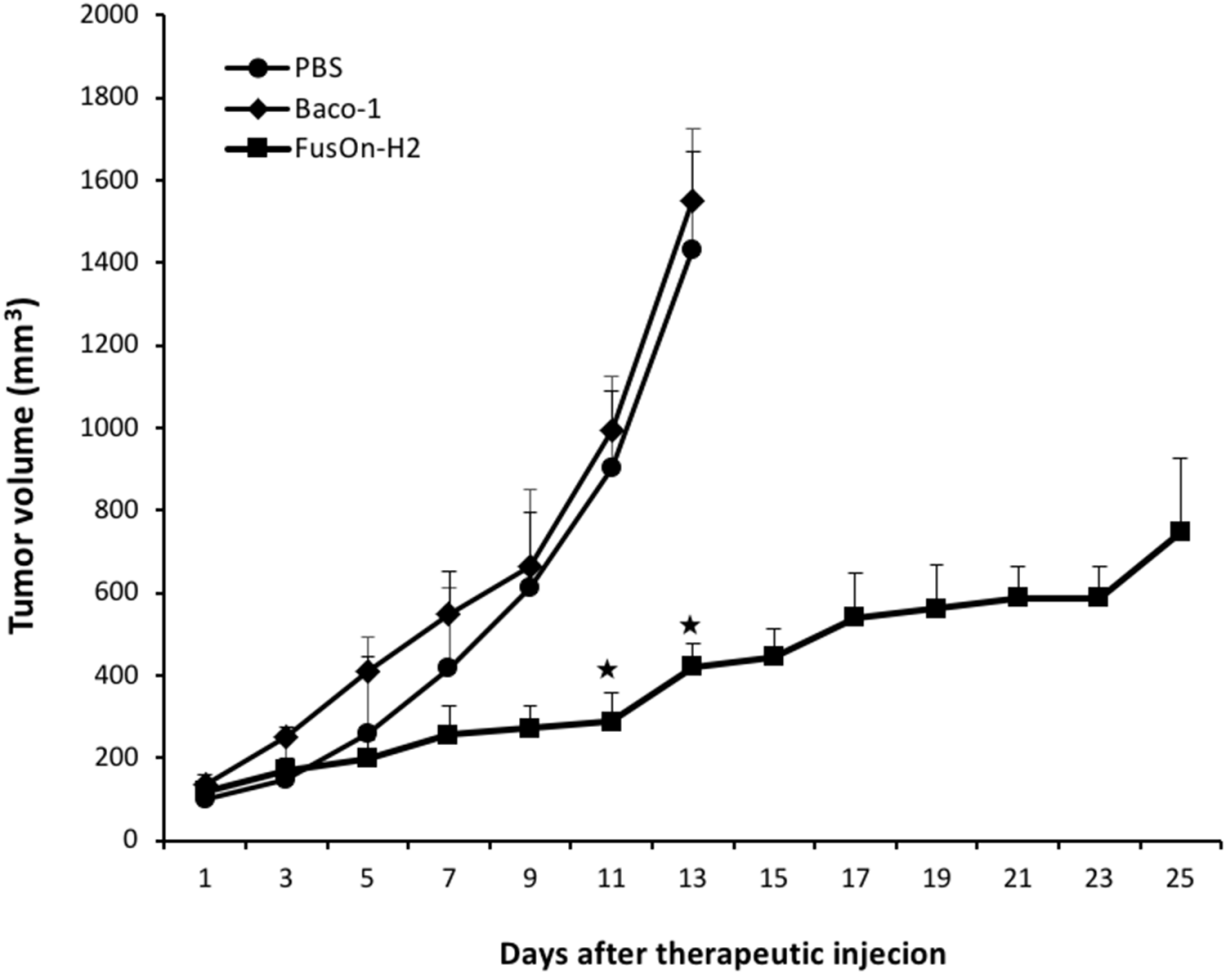
Therapeutic effect of Baco-1 and FusOn-H2 against tumors established from implantation of A375 cells, one of the tumor cells that express nectin-2 but not HVEM or nectin-1 Freshly harvested A375 cells were implanted to the right flank of NOD-SCID mice. Once tumors reached the approximate size of 5 mm in diameter, mice were randomly divided into three groups to receive intratumoral injection of 1×10^7^ pfu of Baco-1 or FusOn-H2, or PBS as a control. Tumor growth was monitored for 25 days (for FusOn-H2 treated group) or until mice had to be euthanized due to the large tumor burden (in PBS and Baco-1 treated groups). ★p<0.05 as compared to the groups treated with Baco-1 and PBS).

To compare the *in vivo* therapeutic effect of Baco-1 and FusOn-H2 against tumors that only express nectin-2 but not HVEM or nectin-1, we implanted A375 tumor cells to the right flank of NOD-SCID mice. Once tumors reached the approximate size of 5 mm in diameter, mice were treated with intratumoral injection of 1×10^7^ pfu of either Baco-1 or FusOn-H2. A third group of tumor-bearing mice was treated with PBS as a control. Tumor size was monitored and the calculated tumor volume was plotted in Figure 6. The results show that the tumors in Baco-1 treated group grew at almost identical rates as in the PBS control, indicating that it showed little or no therapeutic activity against this tumor. By day 13, the tumors in both Baco-1 and PBS treated groups had reached a large size requiring animals to be euthanized. This is in contrast to the results from our previous studies on tumors formed from other cancer cell lines that express HEVM and/or nectin-1, which showed that Baco-1 produced clearly measurable antitumor activities on all these tumors [14–17]. FusOn-H2, by contrast, produced a significant antitumor activity. Tumors in the FusOn-H2 treated group were significantly smaller than those in Baco-1 treated group as early as at days 11, and the difference increased at later times. All animals were still alive by the end of the experiment with tumors in some animals barely detectable. Together, these results suggest that the ability of oncolytic virus to spread cell-cell within tumor tissues plays a pivotal role in its therapeutic activity in both *in vitro* and *in vivo* settings.

## DISCUSSION

The direct oncolytic effect of a virotherapy is mainly determined by two major factors – the ability of the virus to infect the malignant cells and the extent of the virus to spread within tumor tissues. Almost all viruses infect target cells by initially binding to a cellular receptor. As such, the expression level of the receptor dictates the susceptibility of the cells to the infectivity of the virus. Some viruses such as adenovirus use a single cellular receptor that has a restricted expression limited to specific cell populations. Consequently, lack of receptor expression has been one of the frequent obstacles encountered during application of vectors derived from these viruses. In contrast, four cellular receptors have been identified that HSV uses for infection and cellular entry. As such, lack of receptor expression has not generally been considered as a barrier for HSV-based oncolytic virotherapy. Our data suggest the existence of a subpopulation of tumor cells that only express the nectin-2 but not the other receptors, and that only the HSV-2 (FusOn-H2) but not HSV-1 (Baco-1) based oncolytic virus can spread cell to cell in these tumor cells. *In vivo* studies show that tumors formed from one of these cells are almost completely resistant to the oncolytic effect of Baco-1 but remain sensitive to the therapeutic intervention of FusOn-H2. Our data suggest that this subpopulation of tumor cells is intrinsically resistant to oncolytic viruses derived from HSV-1, such as Baco-1 used in this experiment or T-VEC that has been approved for clinical use. Conceivably, therapy resistance could develop during HSV-1 based virotherapy through selecting tumor cells of this subpopulation. Our data suggest that HSV-2 based oncolytic viruses such as FusOn-H2 could be used to treat the resistant tumors should they develop during virotherapy with HSV-1 based oncolytic viruses such as T-VEC.

Previous studies show that nectin-2 has a higher entry activity for HSV-2 and is an inefficient receptor for HSV-1 except for some variants that contain certain mutations in gD [12]. Our data show that Baco-1 and FusOn-H2 can readily infect this subpopulation of tumor cells. However, our data show that the major difference lies in their ability to spread from cell to cell instead. While Baco-1 failed to show any sign of cell to cell spread in any of these tumor cells, the spreading of FusOn-H2 in these cells does not seem to be significantly impeded. Our demonstration of the inability of Baco-1 to spread in these cells is consistent with the report by Cocchi et al., in which they show that nectin-1, but not nectin-2, facilitates cell to cell spread of wild type HSV-1 [18]. The fact that FusOn-H2 can spread in these tumor cells indicates that HSV-2 gD can use nectin-2 for both entry and cell to cell transmission.

It is interesting to note that nectin-2 has been reported to facilitate the entry and cell to cell spread of fusogenic phenotypes of HSV [19, 20], and FusOn-H2 has the syncytial property. Nevertheless, our data show nectin-2 is sufficient to mediate cell to cell spread of FusOn-H2 even in A375 cell in which it does not show a clear syncytial phenotype. Regardless, the ability of FusOn-H2 to rely on nectin-2 alone for both infection and spread renders this HSV-2 based oncolytic virus with the capability to produce satisfactory therapeutic effect against this subpopulation of tumor cells that would otherwise resist to the oncolytic effect of an HSV-1 based virotherapy. Likewise, this unique ability of FusOn-H2 may thus lead to less therapy-resistant tumor formation than virotherapy with an HSV-1 based oncolytic virus in clinical applications on cancer patients. It may also be possible to utilize HSV-1 derived vectors containing the gD mutation that binds nectin-2 to create vectors that overcome such resistance [21].

As compared with a standard gene therapy approach, one of the major differences of virotherapy is the ability of the oncolytic virus to spread from the initially infected tumor cells to the surrounding tumor cells. Our data reinforce the pivotal rule of cell-cell spread in contributing to the overall antitumor activity of cancer virotherapy. In essence, our data demonstrate that Baco-1 can infect this subpopulation of tumor cells at almost the same efficiency as FusOn-H2. Moreover, the yield of Baco-1 is substantially higher than that of FusOn-H2 in these tumor cells. Surprisingly, the killing effect of FusOn-H2 was more superior to that of Baco-1 when measured *in vitro*. Most importantly, the *in vivo* studies on an animal tumor model with one of the cell lines from this subpopulation show that Baco-1 is completely ineffective while FusOn-H2 produced a significant therapeutic benefit. Together, these point to the importance on enhancing the cell-cell spreading capability of oncolytic viruses as a desirable strategy to potentiate cancer virotherapy in future research.

## MATERIALS AND METHODS

### Cells and viruses

Hela, A375 and CHO-K1 cells were purchased from American Type Culture Collection (Manassas, VA). Mel526 was obtained from Dr. Navin Varadarajan (University of Houston). EC9706, a human esophageal carcinoma line [22], was kindly provided by Dr. Mingrong Wang (Chinese Academy of Medical Sciences). CHO-N2 cells were derived from CHO-B (a kind gift to TPC from Dr. Patricia Spear, Northwestern University, Chicago, IL, USA), which was established from CHO-K1 by transducing the *nectin-2* gene into the cells. CHO-N2 cells were obtained by sorting CHO-B cells for the high nection-2 expression subpopulation. A375-HVEM was established in our own laboratory by transducing the *HVEM* gene into A375 cells using a lentiviral vector. Baco-1 is a HSV-1 (strain 17) based oncolytic virus and the details of its construction have been described elsewhere [14]. Briefly, Baco-1 was constructed by deleting both copies of the *ICP34.5* gene that functions as a neurovirulent factor during HSV infection [23]. Another activity associated with *ICP34.5* gene is blocking viral replication in nondividing cells [24–26], and as such, deletion of this gene from viral genome has been widely used for construction of HSV-1-based oncolytic viruses. FusOn-H2 is a HSV-2 (strain 186) based oncolytic virus and the details of its construction has been reported [4]. Briefly, it was derived from HSV-2 by deleting the N-terminal region of the *ICP10* gene, which facilitates virus replication in non-mitotic cells [27], and as such, deletion of this region renders the mutant virus with the ability to selectively replicate in malignant cells. Like Baco-1, it also contains the GFP gene in its genome [4].

### GFP visualization of oncolytic HSV infectivity

To visualize the infectivity of both Baco-1 and FusOn-H2, cell monolayers of Hela, A375, A375-HVEM, Mel526, EC9706, CHO-K1 and CHO-N2 cells were infected with the viruses at either 0.1 or 1 pfu/cell. The infected cells were visualized under a fluorescent microscope (Nikon Instruments Inc., Melville, NY) at the indicated time for identification of GFP positive cells

### Flow cytometry measurement on cell surface receptor expression

In order to determine the expression profile of HSV receptors on cell surface, human cancer cell lines Hela, A375, A375-HVEM, Mel526 and EC9706 were separately stained with 5 μl of PE conjugated anti--human HVEM, nectin-1 and nectin-2 antibodies (BioLegend, San Diego, CA) in 2% FBS-PBS at 4°C for 30 min. The stained cells in 2% FBS-PBS were analyzed by flow cytometry (BD Biosciences, San Jose, CA).

### Virus yield and killing activity measurement

To compare virus yields in human cancer cells with different expression profile of HSV receptors, Hela, A375, A375-HVEM, Mel526 and EC9706 cells in 12-well plates were infected with Baco-1 or FusOn-H2 at 1 pfu/cell for one hour. After three times of repeated wash with PBS, the cells were incubated at 37°C for 24 h before they were harvested for determining virus yield by plaque assay on Vero cells.

For comparing the killing activity of the two different oncolytic HSVs, Hela, A375, EC9706 and CHO-N2 cells were seeded in 12-well plates overnight before they were infected with either Baco-1 or FusOn-H2 at 0.1 or 1 pfu/cell, or medium only (as mock-infected controls). Cells were harvested 48 h later and viable cells were quantitated by trypan blue exclusion assay. The percentage of cell killing was calculated by the following formula: (living cells of mock-infected well – living cells of infected well)/living cells of mock infected well x100.

### Animal studies

Six-week old NOD-Scid mice were purchased from Jackson Laboratories (Indianapolis, IN). All animal experimental procedures were approved by the University of Houston Animal Care and Use Committee. A375 cells were cultured in 10% DMEM in standard conditions until 70% confluence. Cells were then trypsinized, pelleted and resuspended in DMEM at a concentration of 2×10^7^ per ml. 2×10^6^ (in 100 μl) were subcutaneously implanted into the right flank of mice. Mice were then randomly divided into three groups. For evaluating the therapeutic efficacy of virotherapy, mice received intratumoral injection of 1×10^7^ pfu of oncolytic viruses (or PBS as a mock control) when tumor reached the approximate size of 5 mm in diameter. Tumor growth was monitored weekly by bidirectional measurements using a caliper, and the tumor volume was calculated by the formula [mm3] = (length x (width)^2^ /0.5.

### Statistical analysis

All quantitative data are reported as mean ± standard deviations (SD). Statistical analysis was made for multiple comparisons using analysis of variance and Student's *t*-test. *p*-value < 0.05 was considered to be statistically significant.
